# Barriers and facilitators to implementing an urban co-responding police-mental health team

**DOI:** 10.1186/s40352-018-0079-0

**Published:** 2018-11-22

**Authors:** Katie Bailey, Staci Rising Paquet, Bradley R. Ray, Eric Grommon, Evan M. Lowder, Emily Sightes

**Affiliations:** 1 Center for Health & Justice Research, Indiana University Public Policy Institute, 334 N. Senate Avenue, Suite 300, Indianapolis, IN 46204 USA; 2 Indiana University – Purdue University Indianapolis, School of Public and Environmental Affairs, 801 W. Michigan Street, Indianapolis, IN 46202 USA; 3 Indiana University – Purdue University Indianapolis, School of Public and Environmental Affairs, Indianapolis, USA

**Keywords:** Persons with mental illness, Co-response teams, Implementation, Police, mental health emergency response, Pre-arrest diversion, Mobile crisis teams, Urban

## Abstract

**Background:**

In an effort to reduce the increasing number of persons with mental illness (PMI) experiencing incarceration, co-responding police-mental health teams are being utilized as a way to divert PMI from the criminal justice system. Co-response teams are typically an inter-agency collaboration between police and mental health professionals, and in some cases include emergency medical personnel. These teams are intended to facilitate emergency response by linking patients to mental health resources rather than the criminal justice system, thus reducing burdens on both the criminal justice systems as well as local healthcare systems. The current study examines the barriers and facilitators of successfully implementing the Mobile Crisis Assistance Team model, a first-responder co-response team consisting of police officers, mental health professionals, and paramedics. Through content analysis of qualitative focus groups with team members and interviews with program stakeholders, this study expands previous findings by identifying additional professional cultural barriers and facilitators to program implementation while also exploring the role of clear, systematic policies and guidelines in program success.

**Results:**

Findings demonstrate the value of having both flexible and formal policies and procedures to help guide program implementation; ample community resources and treatment services in order to successfully refer clients to needed services; and streamlined communication among participating agencies and the local healthcare community. A significant barrier to successful program implementation is that of role conflict and stigma. Indeed, members of the co-response teams experienced difficulty transitioning into their new roles and reported negative feedback from other first responders as well as from within their own agency. Initial agency collaboration, information sharing between agencies, and team building were also identified as facilitators to program implementation.

**Conclusion:**

The current study provides a critical foundation for the implementation of first-responder police-mental health co-response teams. Cultural and systematic barriers to co-response team success should be understood prior to program creation and used to guide implementation. Furthermore, attention must be directed to cultivating community and professional support for co-response teams. Findings from this study can be used to guide future efforts to implement first-response co-response teams in order to positively engage PMI and divert PMI from the criminal justice system.

## Background

Since the 1970’s, persons with mental illness (hereafter referred to as “PMI”) have been managed precipitously by the criminal justice system. One of the commonly cited reasons for the mismanagement of PMI in justice settings is the process of deinstitutionalization that began in the 1950’s. Before this time, the national inpatient rate was approximately three times higher than the national incarceration rate (Raphael & Stoll, 2013). Incarceration outpaced institutionalization in the mid 1970’s, and this gap became increasingly large following the exponential growth in incarceration rates through the 1980’s and 1990’s (Raphael & Stoll, 2013). Today, PMI are three times more likely to be in jail or prison than in a hospital receiving appropriate treatment (Taheri, 2016). Scholars do not know exactly how many incarcerated individuals suffer from mental illness, but estimates range from 14% to as many as 50% (Steadman, Osher, Robbins, Case, & Samuels, 2009; Bronson, & Berzofsky, 2017).

Given the prevalence of PMI, local jurisdictions have started implementing programs aimed at reducing unnecessary arrest and incarceration and linking this population to community-based treatment and supports. Crisis Intervention Training (CIT) is an example of this kind of program for police officers; the CIT curriculum includes a myriad of topics related to behavioral health and substance abuse as well as police officer liability, policies and procedures, and equipment (Dupont, Cochran, & Pillsbury, 2007; Compton, Bahora, Watson, & Oliva, 2008). However, many jurisdictions see CIT as part of a broader “pre-booking” approach to addressing police-PMI relations in the community and have collaborated with local community healthcare providers to create another type of “pre-booking” diversion referred to as *co-responding police-mental health teams*. These teams typically involve partnering a sworn police officer with a mental health or substance abuse professional (Shapiro et al., 2014). Thus, co-response teams go beyond training police officers by integrating officers with trained professionals who specialize in behavioral health problems.

The present study provides insight into the barriers and facilitators of implementing a co-response team in Indianapolis, Indiana, a metropolitan city in the Midwest. This co-response model, the Mobile Crisis Assistance Team (MCAT), provides a coordinated team response by police, paramedics, and master’s level behavioral health clinicians to emergency calls for service involving PMI. In order to study and document program implementation, the MCAT program was piloted in a single Indianapolis Metropolitan Police Department (IMPD) between August 1 and December 9, 2017. Researchers collected qualitative data interviews and focus groups with members of the MCAT (police, paramedics, and clinical social workers) as well as agency stakeholders who developed and implemented the program throughout the first 4 months of the program. In transcribing and coding these interviews we identify themes around implementation of the MCAT and link these findings back to the literature on co-response teams to inform future efforts in developing similar integrated interagency collaboration models.

### Police responses to mental illness

One of the most prevalent specialized police strategies to address PMI been CIT, wherein police officers are trained about mental illness and how to respectfully and safely interact with PMI (Dupont, Cochran, & Pillsbury, 2007; Compton, Bahora, Watson, & Oliva, 2008). The original CIT program was established in 1998 by Memphis Police Department in response to the police shooting of a PMI in crisis (Compton et al., 2008). The main goal of CIT is to divert PMI away from the criminal justice system into responsive mental health services (Watson, Ottati, Morabito, Draine, Kerr, & Angell, 2010). Studies on the effectiveness of CIT have been generally positive (Compton, et al., 2008); however, some research indicates that arrest rates are similar between CIT and non-CIT trained officers, and that positive effects seen are the result of CIT officers referring PMIs to treatment who would have otherwise just been “contacted” but not actually arrested (Watson et al., 2010). This suggests that CIT may be only part of a broader “pre-booking” approach to addressing police-PMI relations in the community.

Recognizing that CIT may just be a part of the solution to managing PMI calls for service, many police departments have collaborated with local community healthcare providers to create another type of “pre-booking” diversion, generally referred to as co-responding police-mental health teams (Shapiro et al., 2015). Many terms are used to refer to co-response teams; these include: mobile crisis intervention teams, crisis outreach and support teams, or ambulance and clinical early response teams. Generally speaking, teams involve a sworn police officer who is partnered with a mental health or substance abuse professional. In some instances, co-response teams also integrate a medical professional (such as a nurse or paramedic) or a peer specialist (such as an individual in recovery from mental illness or substance use disorder) (Hay, 2015). Co-response teams are built on the premise that “the more police and mental health workers collaborate, the better the two systems can serve consumers” (Rosenbaum, 2010). Teams usually have several common goals, including diverting PMI from the criminal justice system, increasing consumer access to mental health and substance abuse treatment, de-escalating crises and preventing injuries, and reducing pressures on the criminal justice and healthcare systems, all while remaining cost effective and accountable (Steadman et al., 2001; Shapiro et al., 2014). Co-response teams also differ in several important ways, both in terms of the responses they provide and the populations they serve. For example, some teams provide an immediate crisis response. That is, they arrive at the scene of a crisis after a call for service has been placed and proceed to operate as first responders, with the assistance of a clinician, social worker, or emergency medical technician. Other types of teams operate as a post-response (or second responders) and arrive after an initial first response has occurred.

The prevalence of police-mental health co-response team implementation in the United States is unknown; in Canada, estimates suggest that these joint teams are the “predominant mobile response” to mental health crises (Kean, Bornstein, & Mackey, 2012). This is concerning, given that there is only a limited amount of research on co-response teams, especially in terms of outcomes and essential components for implementation (Forchuk, Jensen, Martin, Csiernik, & Atyeo, 2010; Boscarato, Lee, Kroschel, Hollander, Brennan, & Warren, 2014). Puntis (2018) and colleagues conducted a systematic review of the literature on co-responder models of police mental health triage, finding nine studies on models based in the United States of 23 total studies identified. The authors acknowledge a lack of literature in this field, particularly regarding model description and fidelity indicators that lead to good practice as well as patient outcomes. However, preliminary research suggests that co-response teams are generally able to attend more crisis runs than regular officers at reduced response times (Baess, 2005; Kisely et al., 2010; Department of Health and Human Services, Victoria, 2012); make fewer arrests and reduce time first responders spend on managing crisis scenes (Lamb, Shaner, Elliot, Decuir, & Foltz, 1995; Steadman, Deane, Borum, & Morrissey, 2000; Baess, 2005; Kisely et al., 2010; Department of Health and Human Services, Victoria, 2012; Shapiro et al., 2014); and minimize burdens on local medical systems by directing fewer crises to emergency care (Baess, 2005; Department of Health and Human Services, Victoria, 2012) and state hospitals (Scott, 2000), suggesting the possibility of cost-effectiveness (Scott, 2000; Rosenbaum, 2010; Department of Health and Human Services, Victoria, 2012). However, several barriers to the development and implementation of a co-response program have been identified. Findings from these studies suggest that the positive outcomes described above are dependent on several factors, including adequate staffing and vehicles (Baess, 2005), sufficient team member cross-training (Baess, 2005), a centralized location for more rapid dispatch (Steadman et al., 2000), and access to pertinent medical and criminal histories about the PMI served (Baess, 2005; Hartford et al., 2006; Shapiro et al., 2014; Kirst et al., 2015). Additionally, there were recurring issues with role clarity and differences in professional cultures between team members, contributing to implementation difficulties (Kirst et al., 2015; Council of State Governments, 2018).

### The current study

A special report for the Mayor of Indianapolis released in October 2016 found that the county’s jail capacity numbers were at a “critical” level, to the extent that some individuals arrested in Marion County were being held in a county jail approximately 160 miles from Indianapolis (BKD, 2016). In response to jail overcrowding and a substantial increase in the jail population between 2015 and 2016, the Mayor formed a task force to study and generate actionable items for reform of the city’s criminal justice system in order to reduce the number of incarcerated individuals. Among other initiatives, the reform plan resulted in the creation of the MCAT pilot program, a co-responding police-mental health team model with the addition of a medical professional. The development of this program was in response to the large numbers of incarcerated PMI nationally, and the fact that PMI are more likely than the general population to be jailed. In the case of MCAT, an IMPD officer partnered with both an Eskenazi Health Midtown (Midtown) mental health clinician and an Indianapolis Emergency Medical Services (IEMS) paramedic. Each agency identified a coordinator within its leadership to design and implement the MCAT program in concert. These official coordinators developed and implemented training, identified potential team members from their respective agencies, and made procedural and logistical decisions with support from the Indianapolis Office of Public Health and Safety. The primary objective of the MCAT pilot was to divert time-consuming, challenging, and complicated pre-arrest situations to a dedicated, specially trained team that could engage, assess, and route individuals to mental health and social services instead of the criminal justice system.

MCAT team members completed approximately 320 h of training together, reviewing topics such as behavioral health, use of force and de-escalation, and legal implications of interagency collaboration among other topics. MCAT teams began responding to crises on August 1, 2017 in a single Indianapolis police district covering approximately 50 mile^2^. This district was selected due to its high Social Disorder Index ranking and high rates of mental/emotional 911 calls and ambulance runs for medical emergencies. Four MCAT units were formed for the pilot project, each working in 12-h shifts, resulting in 24/7 MCAT availability. MCAT members had unique uniforms identifying them as both MCAT personnel and members of their respective agencies. The teams operated out of a non-emergent van with an MCAT logo on the outside. Each team member utilized his or her own laptop to access necessary agency-specific information, although information was shared between team members to facilitate contact and treatment. The roles of MCAT team members were fluid to allow dynamic responses to crises but, generally, the police officer ensured security at the scene; the clinician facilitated mental health assessments and treatment linkage; and the paramedic addressed any medical issues, checked patient vitals, and performed assessments related to substance use symptoms when necessary. Importantly—and distinct from other co-response teams—the MCAT served primarily as a first responder unit, not a post-response unit. Thus, the MCAT responded to the scene of a crisis at the request of other first responders, or self-dispatched upon hearing of a relevant crisis via IMPD or IEMS dispatch radio. However, when available, MCAT also conducted follow-ups with individuals they previously encountered to encourage linkage with services. By the conclusion of the pilot in December 2017, MCAT responded to a total of 566 emergency calls for service with a range of 1 to 11 responses per day. MCAT teams self-dispatched in nearly two-thirds (63%) of runs, while the rest of the runs (35%) were primarily in response to specific requests for help from IMPD. A mental health emergency was the most common event to which MCAT responded, with 59% of runs involving PMI. The second and third most common emergencies to which MCAT responded were overdose or substance abuse related and self-harm related (35% and 34% respectively).

## Method

Qualitative data were collected from two sources during the first 4 months of the pilot period: focus groups with MCAT members and one-on-one semi-structured interviews with key stakeholders. Three members of the research team conducted two semi-structured focus groups with members of the MCAT units. Each focus group consisted of two teams (six MCAT members in each focus group, *n* = 12) and lasted approximately 2 hours each. These focus groups captured the entire population of line-level staff involved in the MCAT effort. A semi-structured guide was used to facilitate these focus groups, allowing for probing, clarification, and contextualization when appropriate (see Appendix 1). The primary goal of focus groups was to understand frontline staff perspectives on implementation, including any perceived barriers or facilitators to successful MCAT operation. A consensus meeting was held after each focus group among research team members and meetings were transcribed. One author conducted interviews with MCAT program developers and stakeholders, which included personnel from IMPD, IEMS, the Indianapolis Department of Public Health & Safety, and Midtown (*n* = 9) (see Table 1). A semi-structured protocol was used to administer these interviews and focused on program development and implementation, including perceived barriers and facilitators to these processes (see Appendix 2). Each interview lasted approximately 1 hour. All of the interviews were audio recorded and transcribed.

**Table 1:** Interview participants

Stakeholder Agency	Stakeholder Title	Primary Role in MCAT Implementation
IMPD	Chief	IMPD buy-in and support of high-level cross-agency collaboration
IMPD	Executive Officer	Oversight of day-to-day MCAT implementation and operation - supervisor of MCAT officers
IMPD	Commander of IMPD East District	Commander of East District police district where MCAT program was piloted. Facilitated cooperation between MCAT and East District officers.
IEMS	Chief	IEMS buy-in and support of high-level, cross-agency collaboration
IEMS	Public Safety Liaison Director	Oversight of day-to-day MCAT implementation and operation – supervisor of MCAT IEMS personnel
Midtown	Clinical Supervisor	Oversight of day-to-day MCAT implementation and operation - supervisor of MCAT clinical personnel
Midtown	Crisis Specialist	With experience on other police/mental health co-response follow-up initiatives, guided development of MCAT model and training
Midtown	Emergency Department Physician	Receives MCAT patients
Indianapolis Office of Public Health and Safety	Director	Implementation of Mayor’s public health and safety reform initiatives including MCAT - coordinate public safety agencies and collaboration with Midtown.

### Analytic strategy

Transcribed qualitative data were reviewed and independently coded by three members of the research team. Coding followed a grounded theory approach (Strauss and Corbin 1998; Charmaz 2001) in which researchers looked at general patterns in the data and then proceeded to identify coding categories. Using directed content analysis to focus the research findings (Weber, 1990; Hsieh & Shannon, 2005), coders classified emergent themes within the broad categories of “barriers” and “facilitators” to MCAT development and implementation. To establish initial inter-rater agreement, researchers independently coded three qualitative data sources using NVivo qualitative analysis software (v.10, QSR International 2014). Then the research team met to discuss emerging themes that originated from the pilot round of coding and collaboratively developed a final coding procedure for all of the qualitative sources. Coders agreed upon categorical titles to describe each common theme (referred to as “nodes” or “cases” in NVivo) and discarded themes that were not corroborated by all three coders. Where coding categories slightly differed amongst researchers, these were discussed with the full research team and, through intersubjective agreement, were folded into broader conceptual categories. For example, a “technology” child node under the mother node of “facilitators” was folded into the broader category of “information sharing.” Upon individually coding all qualitative materials using the agreed upon coding procedures, researchers met a final time to review and establish final categorical titles for the major themes gleaned. Major themes reported here are those independently identified by all three coders and include topics reported by four or more stakeholders from at least two different agencies across the three sets of qualitative data collection. Frequencies were computed for each coding category and source using NVivo.

## Results

Table 2 provides a list and brief description of the barrier and facilitator related themes that emerged during the coding process. Below we outline each of these themes in greater detail and provide examples from the data that illustrate the specific barrier or facilitator.

**Table 2:** Barriers and Facilitators of MCAT Implementation

Barriers	Description
Policies and Procedures	A lack of clear policies and procedures led to confusion and inconsistency among MCAT units.
External Coordination	MCAT stakeholders fell short of successfully coordinating with outside agencies service the same population.
Treatment Resources	A lack of local treatment facilities complicated diversion into treatment.
Role Conflict and Stigma	Some MCAT members struggled transitioning into their new roles on a collaborative, mental health-focused team.
	Oversight of day-to-day MCAT implementation and operation – supervisor of MCAT IEMS personnel
Facilitators	
Initial Citywide Collaboration and Buy In	MCAT implementation was bolstered by multiple city agencies who collaborated closely to develop the program.
Information Sharing	Triangulation of MCAT consumer information was integral to the program’s operations.
Team Building	Team building exercises during initial training laid a solid foundation for three person teams going forward.

### Policies and procedures

The MCAT was established as a pilot program with little oversight or guidance in how teams would operate. While this flexibility allowed team members to continuously learn and adjust to real-time needs in the community, a lack of clear direction sometimes led to confusion, variation in crisis response, and frustration among the teams. As one team member noted,

“We need a clear mission statement; we don’t know whether we’re supposed to be responding to certain runs or not. Right now, we’re all taking different approaches to these calls. What is our role supposed to be? Because I don’t think we have a clearly defined role.”

However, MCAT leadership was hesitant to limit the ways in which units could respond; preferring instead for discretion with the understanding that the pilot program would reveal the ways in which MCAT could be most useful. As one leader explained, “We didn’t want to make everything black and white for them, because we want them to keep thinking. To make decisions on their own.” Another leader described what would happen if the pilot program was rolled out with strict policies and procedures already in place,

“I think putting too many bright line rules would probably be detrimental to it. Because then it’s like when you have these bright line rules and officers are held accountable to these rules and they could get in trouble and the way you don’t get in trouble is to not deal with it at all… and we don’t want that.”

### External communication

Inter-agency communication can be difficult when combining multiple agencies into one emergency response initiative, particularly around the topics of mental health and substance use. IMPD, IEMS, and Midtown experienced learning pains in establishing internal communication protocols and sharing or coordinating resources; however, communicating and coordinating MCAT goals and responsibilities with external actors in the community was a more salient barrier to implementation. For example, several team members expressed frustration at being asked who they were and being confused with other agencies in the area doing similar work, stating, “People don’t know who MCAT is or what they do.” Participants described a host of examples of how other first responders struggled to understand their role: “Firefighters make comments like, ‘No one else does it like this’, and that’s frustrating.” Team members lamented there was no dedicated liaison to communicate MCAT’s role, stating, “It’d be nice if higher ups could go to places out in the community and introduce MCAT, explain what we do.”

MCAT leadership acknowledged that operations were not adequately publicized or well integrated into the local network of first responders. These disconnects were especially prominent between officers and other agencies’ first responders. One leader conceded,

“[MCAT leadership] probably could have communicated it a little better amongst supervisors and officers… so they had a better idea. There was a little confusion about what the responsibilities were. And there were officers that really didn’t know the [MCAT teams] were out there.”

Similar statements were made regarding MCAT coordination with community healthcare providers outside of MCAT partner Midtown. MCAT members were skeptical of that their roles were clearly communicated to the hospitals they work with, stating, “[Key stakeholders] should have met with other hospitals before the MCAT program started because they need to know who we are and why we’re bringing in patients.” Others agreed, “Most people don’t even know what MCAT is, so I don’t even think that most [doctors] would realize that their patient was brought by MCAT to the emergency department.”

There was also a perceived need to better market MCAT operations to the public in order to differentiate this program from other initiatives that serve the same populations. One stakeholder explained that other community initiatives had misconceptions about what MCAT would and would not do which led to miscommunication. Several MCAT leaders and stakeholders explained and gave examples of this lack of coordination. For instance, existing public safety programs to assist with homeless outreach, emergency crisis follow-up and mental health interventions were not incorporated into the MCAT planning process or implementation, although these efforts serve similar populations to those of MCAT.

Across these subthemes of external communication, it is clear that MCAT operations were not well coordinated with or communicated to other first responders, healthcare providers, or other strategic initiatives in operation across the city.

### Treatment services

The difficulties presented by a lack of treatment services for program consumers surfaced multiple times throughout the research literature on co-response teams (Lamb et al., 1995; Scott, 2000; Steadman et al., 2000; Steadman et al., 2001; Ratansi, 2004; Baess, 2005; Hartford et al., 2006; Saunders & Marchik, 2007; Kisely et al., 2010; Rosenbaum, 2010; Department of Health and Human Services, Victoria, 2012; Kirst et al., 2015), and unfortunately this barrier existed for the MCAT as well. One MCAT stakeholder stated,

“This is the biggest fear for me; you do all of this work on the front end, but there are no real services on the back end, so these people aren’t getting the help, because there are not enough beds or mental health professionals that will work with them, or they don’t have insurance.”

Addressing the needs of populations with behavioral health and co-occurring substance use issues goes well beyond responding to crises and seeking to find avenues for diversion. MCAT team members provide referrals, guide individuals to resources, or transport patients to a hospital or other crisis center, but this does not guarantee that individualized treatment needs will be met. Treatment services may not be accessible and in some instances may simply not exist. Another MCAT stakeholder grimly suggested, “The city was under the impression that there are places to take people; there are not.”

The lack of available outpatient treatment services led participants to rethink the initial build-out of the MCAT program. Rather than emphasizing relationship building across agencies and delivering training to teams as one of the initial steps to planning the implementation of the MCAT program, stakeholders wished more effort would have been placed on inventorying and expanding treatment services. As a MCAT stakeholder suggested,

“If we are talking about launching this in a thoughtful way in other places, then making sure that treatment resources are available [is crucial]. You sort of have to work backwards: if you’re going to go out and find people who need help, you probably want to have that help available.”

Another leader seconded this view, proposing that,

“A lot of time and effort and money is being spent on innovative programs when really probably a lot more time and effort and energy should be spent building the capacity of our behavioral health system to take care of people.”

Limited treatment resources also have the potential to foster frustration and burnout for team members. One MCAT team member expressed exasperation, stating, “People come out of [the hospital] not making changes because they were just given a pamphlet, not services.” Unfortunately, addressing these issues requires bolstering a broader system of behavioral health care beyond the purview of co-response teams, follow-up units, emergency departments, or first responders.

### Role conflict and stigma

Police officers and paramedics had a difficult time transitioning from traditional responsibilities to new roles in a special unit and experienced stigma and negative feedback from within their respective agencies. For example, several team members recounted struggles with other police officers, stating, “[Other officers say] we’re jokes now; we aren’t the real police”. Other team members explained experiencing similar issues with the fire department: “Firefighters have been particularly resistant to understand what we do.” One team member suggested that emergency services was problematic, as well: “Every ambulance I’ve run into thinks we are there to do their legwork.” As a result, some MCAT members had difficulty adjusting to the new identity that was placed upon them. As one clinician explained, “Three different people with three different roles do one job now, not exactly the job they used to do. In a way, you’re giving up your expertise in the spirit of collaboration.”

This difficulty was most prominently manifested in concern about the MCAT uniforms, especially when it came to police officers. As officers explained, “There is honor in your uniform and this MCAT uniform is a halfway-police officer uniform.” One officer stated, “Being clearly identified as a police officer is important to me. Being clearly identified is important for all the roles. I’ve been mistaken for animal control, a mall cop, and Brinks.” Another officer confided, “My biggest hang-up is still the uniform. I want to be more clearly identified as a police officer, and if that is not addressed in the program, it could make or break my decision to stay on the team.”

Leadership anticipated that the transition to a new, non-traditional service role would contribute to team member dissatisfaction. One leader explained how this experience might be affecting officers on teams:

“One of the most difficult things is coming out of what they are normally doing... They have all been on the street... so I think that’s an adjustment for them. Any time law enforcement leaves the first position (street officer)… it’s often difficult to make that transition.”

Leadership also recounted specific issues from the beginning of the pilot,

“There was a lot of pressure and push back, just culturally within the organization. Oh, why are you going to a special unit? What are you even going to be doing? And [officers] couldn’t really answer that... It takes a certain type of officer with a lot of self-confidence and an open mind.”

Despite the stigma that officers were experiencing, leadership insisted that the MCAT uniforms served a very specific function related to the population that MCAT serves. An MCAT stakeholder explained,

“There is a different reaction to [a police officer] in a uniform… They aren’t going to talk to me as much as they would you about their mental health or their drug abuse. And so, my guess is that’s the reason and that would be the pushback I think to go back to their regular uniforms.”

MCAT leadership and stakeholders hold that the MCAT’s more neutral appearance, despite team member misgivings, is critical to the unit’s behavioral health agenda. These themes also demonstrate strains between MCAT leadership and team members who may have different perspectives on how the MCAT program should operate.

### Initial agency collaboration

The City of Indianapolis was largely responsible for spearheading the MCAT pilot program and had previously coordinated efforts between public safety agencies and mental health providers. As one MCAT stakeholder explained,

“I think what made it easier is that Marion County has had a long lasting relationship… even before [MCAT], we had a good relationship with medical services here, a good relationship with Eskenazi, a good relationship with mental health workers. So, from the top down, so there had been a lot of history and a lot of associations with, um, individuals who have thought the same way.”

Respondents especially praised support from the Mayor’s Office, explaining how it provided a platform for resource negotiation and agency accountability. Another stakeholder described his experience during MCAT development similarly:

“It has to start from the top down because this is an ask from everybody... There has to be a return on investment for everybody involved that’s not money...So we have all of these different moving parts, and when you put the leadership together in a collaborative way, and these folks all want to solve the same problem, we can sort of understand, ‘Okay, I can lose over here if I win over here.”

MCAT was able to take advantage of already-existing synergies between agencies to bring agency representatives to the table and initiate dialogue to build the MCAT program. Leadership was open to leaving their agency “silos” to tackle shared problems and had experience working with one another. Further, the high-profile status of the program and support from city-county government may have helped to alleviate initial resource equity concerns.

### Information sharing

One of the most salient facilitators of implementation noted by MCAT members and stakeholders was the ability to share information within the bounds of regulatory and legal mandates. MCAT units believed their “triangulation” of agency information resources results in a greater ability to locate, serve, and follow-up with a specific patient in and after the moment of crisis. Multiple stakeholders throughout the evaluation expressed the importance of information triangulation, and how “combining the systems and software of three agencies creates a powerful tool” for the MCAT.

Upon dispatching to the scene of an emergency, officers and clinicians can independently and respectively compare criminal justice and healthcare records on their laptops to better prepare the team for response. This triangulation helps teams anticipate potential hazards and allows them to reconnect patients with treatment services they may have received in the past. As one MCAT team member explained,

“We can look at [a situation] from multiple different angles. We typically try to do our homework when we go out and see somebody, especially if we have a name ahead of time… The police officer will go and look at what their record is. Our clinician can look to see if they are in the Midtown system, to see if they have been there before... and then from the medical side we can see... how many times they have called in the past few years and now we are putting together a better picture on things.”

An MCAT stakeholder noted the difference these information systems make on operations, particularly noting new abilities to directly observe the sequential outcome of their intervention:

“There is a Midtown clinician that...is able to link [a patient] back to their treatment team via our own medical record and communicate that this person is having a problem, and then you can go in and see [if] this person is getting a follow up from the treatment team the next day, versus kind of just letting them go.”

Combining information from multiple agencies also enabled team members to identify frequent “fliers” or “super utilizers” of local justice system and emergency services. While these individuals had been in contact with all three MCAT agencies prior to the MCAT pilot, the information shared across agencies helped team members to understand the frequency and time lags between contacts. In turn, this information was used to coordinate and deliver an intervention that may be most appropriate to an individual’s current circumstances.

### Team building

Participants acknowledged that one of the most useful aspects of the initial MCAT training was the ability for team members to learn about one another, adopt useful skills from one another, and build relationships. Team members were introduced to the philosophies, language, and procedures of the other agencies during a seven-week training. As one MCAT stakeholder described, “It was apparent that each different agency needed to be a little more aware of the other agencies in order to work more closely together.”

Team members expressed that “the training brought this unit together and I think all the teams are functioning pretty good.” MCAT members emphasized that being able to select their teams was an important element of the initial implementation of the program: “If we were assigned teams, we may not have been as successful, but we got to pick our teams and get along better for it.” Team members spoke of collegial working relationships and noted examples of working styles or approaches drifting across team members. For instance, one clinician complimented his paramedic teammate on subtle changes in the paramedic’s interactions with PMI, citing the paramedic’s willingness to adopt attitudes and behaviors more typical of a clinician than a paramedic. These kind of scene processing and PMI interaction changes were identified by other IMPD and IEMS members of the MCAT, stating that their “mindset was changed because of the training.”

## Discussion

PMI are disproportionately represented in the criminal justice system and, police are often positioned to be the first responders in calls for service for PMI in crisis. Co-response teams have emerged as one potential intervention that could help stem the cycle of criminal justice involvement among PMI. This study examined the Indianapolis MCAT pilot program—a co-responding police-mental health team aimed at pre-arrest diversion—and identified a number of important barriers and facilitators to the program’s implementation.

In terms of barriers, ambiguous policies and procedures were difficult for MCAT members to adapt to in their new roles. Coordination of agencies contributing to the MCAT program and word-of-mouth efforts made to advance MCAT’s objectives and roles were not necessarily being heard by agencies directly or indirectly assisting PMI, which compounded confusion about the program in general and appeared to translate directly to role ambiguity among its line members (Department of Health and Human Services, Victoria, 2012). Developing a systematic set of procedures and resources may increase consistency between teams and reduce confusion for teams trying to decide where to respond, what actions to take, and how to follow up (Department of Health and Human Services, Victoria, 2012). After implementation, policies can and should be reevaluated to determine whether or not they are in line with the program’s ultimate mission or goal. Indeed, leadership acknowledged that some initial flexibility is advisable but that more formalization and guidance is necessary as operations evolve over time. Findings suggest that first responders value the structure associated with clear policies and procedures as a way to effectively carry out job tasks, reduce concerns related to liability, as well as legitimize their work (Kinnaird, 2002).

Stigma emerged as a noteworthy barrier to implementation and was part of a larger issue related to role clarity; this is consistent with the extant literature which suggests that on co-response teams roles, missions, and beliefs do not always align (Scott, 2000; Steadman et al., 2000; Hartford et al., 2006; Saunders & Marchik, 2007; Kisely et al., 2010; Rosenbaum, 2010; Department of Health and Human Services, Victoria, 2012; Kirst et al., 2015). Police in particular felt ostracized by fellow first responders. Indeed, police culture characterized by masculinity and solidarity may be partially to blame for this stigma (Workman-Stark, 2017). Once co-response team members have been identified and assigned, efforts must be made to support and retain these members through in-service and off-service training. Stigma between police officers can be reduced through cultivating a collaborative approach to police work that places higher value on diverse officer skill sets and rewards officers who volunteer to participate in innovative or otherwise non-traditional operations, such as co-response teams (Workman-Stark, 2017).

Despite these barriers, the findings offer several important facilitators to build out and implement first-responder co-response teams. For example, information sharing (or “triangulation”) was one of the most salient facilitators to both the MCAT in this study and other co-response teams (Lamb et al., 1995; Scott, 2000; Steadman et al., 2000; Ratansi, 2004; Baess, 2005; Hartford et al., 2006; Saunders & Marchik, 2007; Kisely et al., 2010; Rosenbaum, 2010; Department of Health and Human Services, Victoria, 2012; Kirst et al., 2015). New forms of data and lenses by which these data could be interpreted appeared to serve as a reinforcement mechanism that enabled team members to understand PMI experiences, track progress, and to try new solutions or those that were perceived to have previously worked. These findings highlight the importance of validating team members’ role by equipping them with the tools necessary for successful job performance, thus increasing self-efficacy. Research indicates high levels of self-efficacy among first responders not only improves their mental health but also increases job satisfaction related to helping others through their work (Pietrantoni & Prati, 2008). Future implementations should seek to develop and coordinate data sharing agreements among co-response team agencies well before implementation. Furthermore, future interventions may wish to move beyond agencies involved in a co-response team and pursue opportunities to share or remotely access non-private justice, health, and treatment system information across geographic areas.

Study findings also highlight the underlying need for increased funding and resource allocation for outpatient mental health treatment options. Indeed, according to Mental Health America, 24% of adults in Indiana have unmet mental health needs, largely due to lack of insurance as well as lack of various treatment types and providers (Mental Health America, n.d.). The difficulties presented by a lack of treatment services for program consumers surfaced multiple times throughout the research literature on co-response teams (Lamb et al., 1995; Scott, 2000; Steadman et al., 2000; Steadman et al., 2001; Ratansi, 2004; Baess, 2005;Hartford et al., 2006; Saunders & Marchik, 2007; Kisely et al., 2010; Rosenbaum, 2010; Department of Health and Human Services, Victoria, 2012; Kirst et al., 2015), and unfortunately this barrier existed for the MCAT as well. An increase in mental health services would facilitate co-response team success as clients are referred to and receive treatment. Future co-response team pilots may wish to integrate time and resources into inventorying available mental health service providers and providers’ entry points well before implementation.

Despite these findings and contributions to the literature this study is not without limitations. First, this was largely a formative evaluation in which we examined the MCAT as a proof-of-concept; that is, can the intervention be implemented and what are some of the barriers and facilitators towards successful delivery of services. As such this study did not examine outcomes or have a basis of comparison to standard responses or interventions serving similar residents. Second, despite some overlap with the available research literature, key themes identified in this study may be a function of the local setting. Future multisite research that enables comparisons of first responder and post-response co-response teams as well as the relative effect of these teams in jurisdictions with and without well-coordinated mental health service providers are needed. Third, although we collected data from all of the MCAT team members and stakeholders, the collection was cross-sectional. Thus, we could not examine changes in attitudes or perceptions over time. Ideally future co-response team research should integrate pre and post surveys to examine changes in attitudes toward PMI and agency collaboration. These data collection would help to move the co-response literature toward more generalizable findings that can help to pinpoint problematic implementation issues and test efforts to mitigate their effect on operations.

## Conclusion

Despite limitations, the current study provides a critical foundation for the implementation of police mental health co-response teams. Cultural and systematic factors that may temper the anticipated benefits of co-response teams should be understood prior to team creation and be used to guide implementation. One key conclusion to take away from this research is the need for collective support for co-response teams from all involved agencies as well as the general community. As co-response team members transition into their new roles and take on new responsibilities, it is critical they have the tools and resources necessary to effectively impact their community and help those living with mental illness. The current study supplements the limited co-response literature that exists by further identifying barriers and facilitators to co-response team implementation. The lessons learned from this study can be used to inform future implementation efforts in order to positively engage PMI and divert them from the criminal justice system into needed services. Additionally, other types of cross-agency collaborations may benefit from the findings of this study as police join with other specialists to divert those in need of treatment from the criminal justice system. For example, recent national efforts between police and treatment providers to divert persons with substance use-related emergencies into recovery services may encounter similar barriers and facilitators to cooperation.

## Appendix 1

### MCAT Semi-Structured Focus Group Protocol

1. First we want to get your general impressions of how MCAT is going
  - So, how are things going?
  - What are your initial perceptions on the usefulness of the MCAT program?
  - Have you come across anything that you weren’t expecting or weren’t fully prepared for?
  - How would you describe the roles [responsibilities] of each person on the MCAT team?
    [PROBE: Would you say that these roles were pre-determined or something that’s developed while working together?
2. Next we want to talk about the decision-making process
  - Can you describe what a “typical” MCAT dispatch process looks like so far?
    [NOTE: if they cannot describe typical dispatch, follow up with what looks similar across calls].
  - Were decision-making protocols ever discussed in training or MCAT development?
    [PROBE: If so, describe].
  - Do you feel like everyone contributes to decisions made at the scene or is there a designated leader?
  - Have you had any challenges related to decision making?
3. Next we want to discuss any barriers and facilitators to the M-CAT
  - What have you found to be a barrier or challenge to responding successfully to M-CAT runs?
  - Are there any specific resources that M-CAT needs to be successful?
  - Is there anything specific that is a barrier towards successfully doing your job once you arrive on the scene?
  - What have you found particularly helpful in responding successful to M-CAT runs?
  [PROBE: Examples might be communication, training, technology].
  e. Thinking about other areas that might implement the M-CAT, is there anything you think should be a “best practice” or a “standard procedure”?
4. Finally we want to discuss what happens after an MCAT run
  - What typically happens to someone after an MCAT involvement?
  [PROBE: Does someone follow up? Always or just in some circumstances?]
  IF YES: What does follow-up look like? Describe this
  b. What do you think the main benefit is to being a person who has a response from the MCAT versus a traditional police response?
  c. Do you think that persons who involved in MCAT runs are less likely to be arrested than those who are involved in a traditional police response?
  [PROBE: Why or why not?]
  d. Is there an MCAT debrief session?
  IF YES: do these inform future runs?

## Appendix 2

### MCAT Semi-Structured Interview Protocol

1. First, could you describe the need that the M-CAT was created to address?
2. From your understanding, could you provide a brief overview of how the program was coordinated?
  - How did stakeholders coordinate/make decisions?
  - What was successful or unsuccessful in this?
3. What barriers to starting the M-CAT program existed?
4. What actions, if any, were taken to overcome those barriers?
  - Do you think they were successful?
5. Is there anything you would have done differently to launch M-CAT knowing what you know now?

***Post-Launch***

6. How do you think the M-CAT program is coming along?
  - In your perception, is it successful?
7. Are there any barriers to a successful MCAT program that have come up since the launch?
8. Do you perceive any necessary course-corrections that the leadership can implement?
  - Do you perceive anything that works particularly well that should be maintained?
  - How would you do this?
9. How do you think the MCAT is perceived by external groups?
10. How do you think the MCAT is perceived generally in your organization?
11. Do you think public or internal perception impacts the ability for MCAT to succeed?
12. For anyone wanting to implement a similar model, what would you say the key components of a successful M-CAT are in terms of:
  - Multi-agency, multi-organizational coordination
  - The Team
  - Equipment
  - Support for ongoing success
  - Other?
13. Is there anything else you would want future implementers of MCAT programs to know?

## Abbreviations

CIT: Crisis Intervention Training
IEMS: Indianapolis Emergency Medical Services
IMPD: Indianapolis Metropolitan Police Department
MCAT: Mobile Crisis Assistance Team
PMI: People with Mental Illness
